# Supplementary material for: A Green-emitting Fluorescent Probe Based on a Benzothiazole Derivative for Imaging Biothiols in Living Cells
Source: Molecules. 2019 Jan 23;24(3):411. doi: 10.3390/molecules24030411 (PMC6384771; doi:10.3390/molecules24030411)
Supplement: Supplementary file 1 [file molecules-24-00411-s001.pdf]

Supplementary Information

A green-emitting fluorescent probe based on benzothiazole derivative for imaging thiols in living cells

Xiaohua Ma [1,2], Yuanqiang Hao [2,*], Guoguang Wu [1,*], and Lin Liu [3,*]

[1] School of Chemical Engineering and Technology, China University of Mining and Technology, Xuzhou 221000, China; mayingjie518@sohu.com (X.M.); b12040004@cumt.edu.cn (G.W)

[2] Henan Key Laboratory of Biomolecular Recognition and Sensing, College of Chemistry and Chemical Engineering, Shangqiu Normal University, Shangqiu 476000, China; haoyuanqiang@aliyun.com (Y.H.)

[3] Key Laboratory of New Optoelectronic Functional Materials (Henan Province), College of Chemistry and Chemical Engineering, Anyang Normal University, Anyang 455000, China; liulin@aynu.edu.cn (L.L.)

* Correspondence: haoyuanqiang@aliyun.com (Y.H.); b12040004@cumt.edu.cn; liulin@aynu.edu.cn (L.L.), Tel.: +86-0370-3112844 (Y.H.)

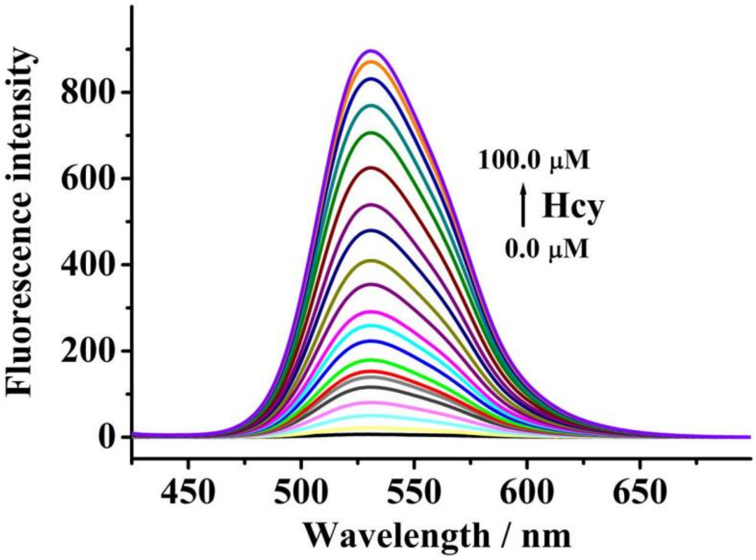

Figure S1 Fluorescence spectra changes of probe **1** (5.0 μM) upon the addition of Hcy (0.0–100.0 μM) in PBS buffer(λ_{exc} = 413 nm, and t = 15 min).

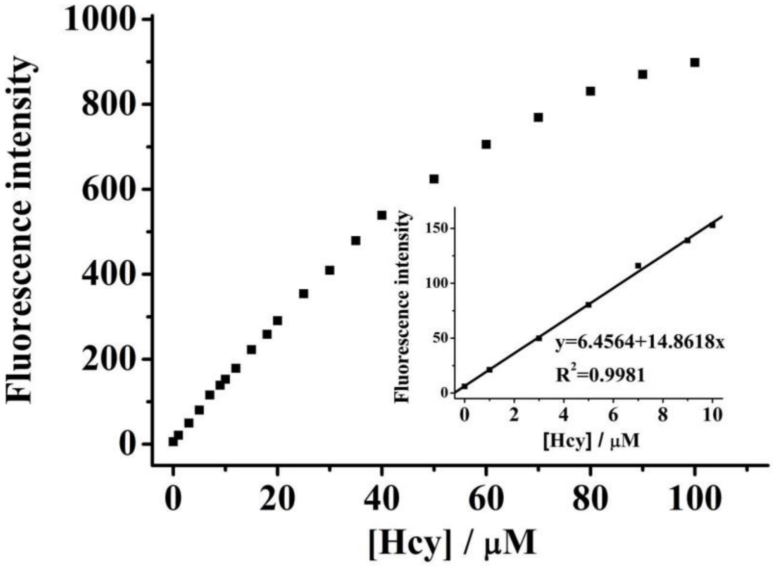

Figure S2 Fluorescence intensity of probe **1** (5.0 μM) at 530 nm as a function of Hcy concentration (0.0–100.0 μM) in PBS buffer (λ_{exc} = 413 nm, and t = 15 min). Inset: the linear relationship between fluorescence intensity and Hcy at low concentrations.

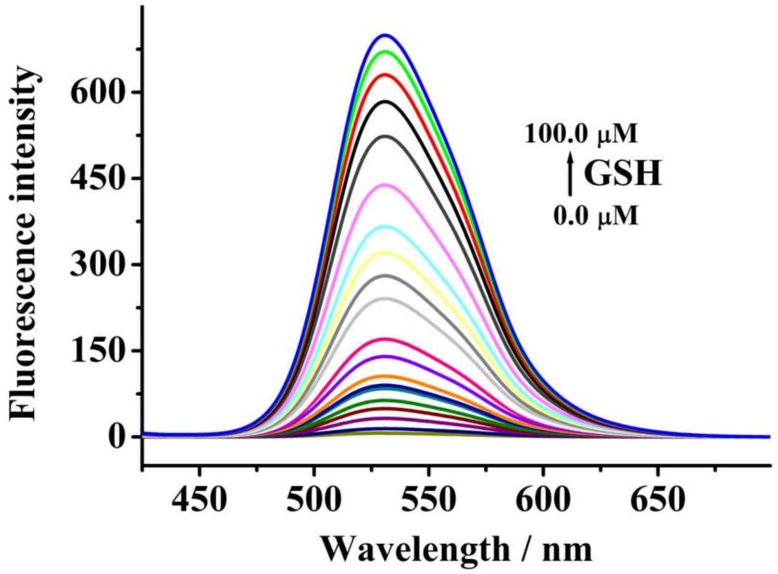

Figure S3 Fluorescence spectra changes of probe **1** (5.0 μM) upon the addition of GSH (0.0–100.0 μM) in PBS buffer (λ_{exc} = 413 nm, and t = 15 min).

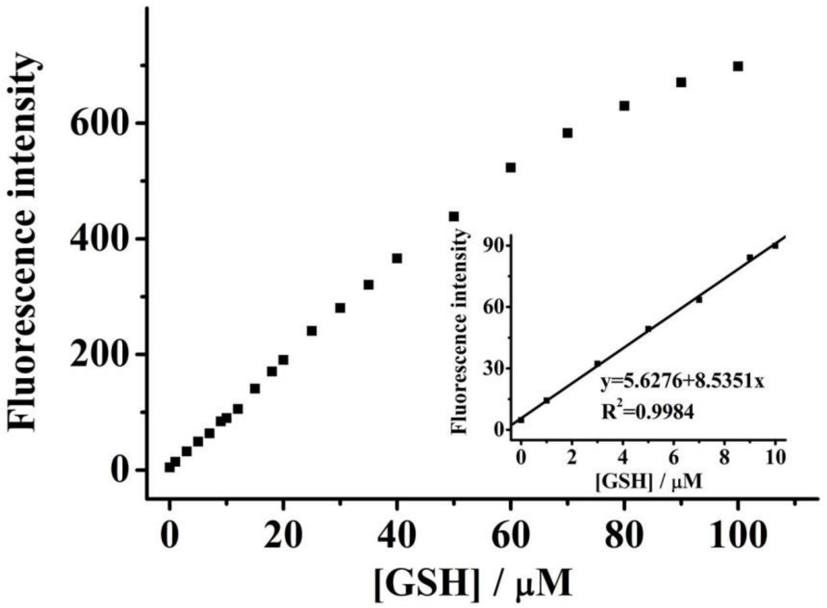

Figure S4 Fluorescence intensity of probe **1** (5.0 μM) at 530 nm as a function of GSH concentration (0.0–100.0 μM) in PBS buffer (λ_{exc} = 413 nm, and t = 15 min). Inset: the linear relationship between fluorescence intensity and GSH at low concentrations.

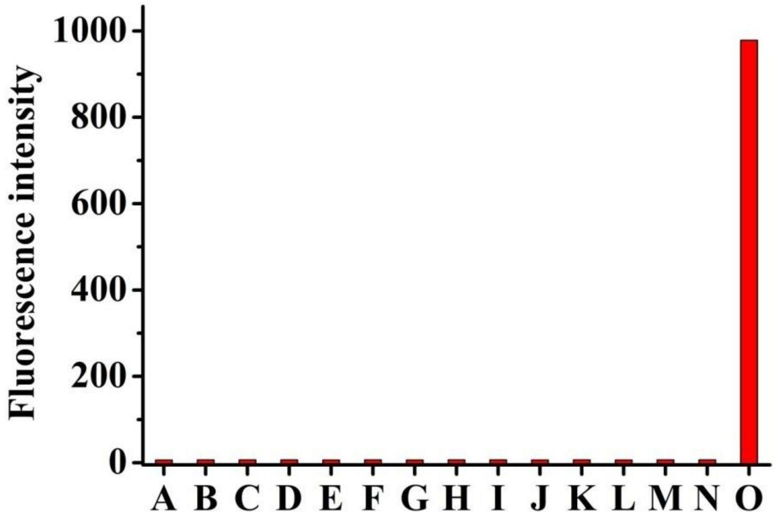

Figure S5 The fluorescence intensity at 530 nm of probe **1** (5.0 μM) upon the addition of the various common amino acid (A-O: 100.0 μM for K^+, Na^+, Mg^{2+}, Ca^{2+}, Zn^{2+}, Fe^{3+}, SO_4^{2-}, SCN^-, AcO^-, CO_3^{2-}, NO_3^-, PO_4^{3-}, NO_2^-, H_2O_2, Cys) (λ_{exc} = 413 nm, and t = 15 min).

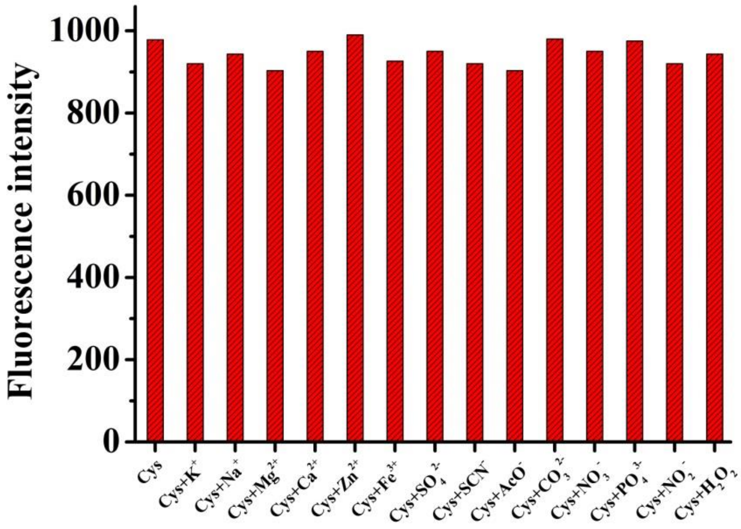

Figure S6 The fluorescence intensity at 530 nm of probe **1** (5.0 μM) to Cys (100.0 μM) with the competition analytes in PBS buffer(λ_{exc} = 413 nm, and t = 15 min).

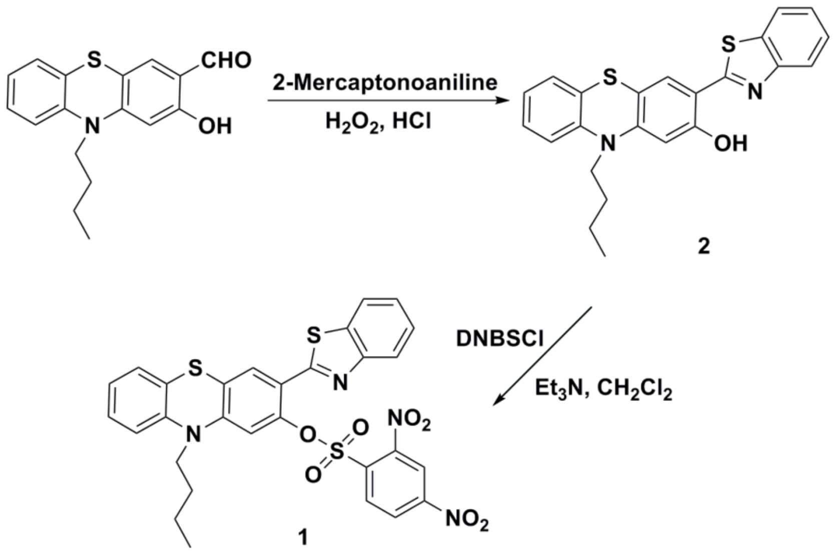

Figure S7 Synthetic route to probe **1**.

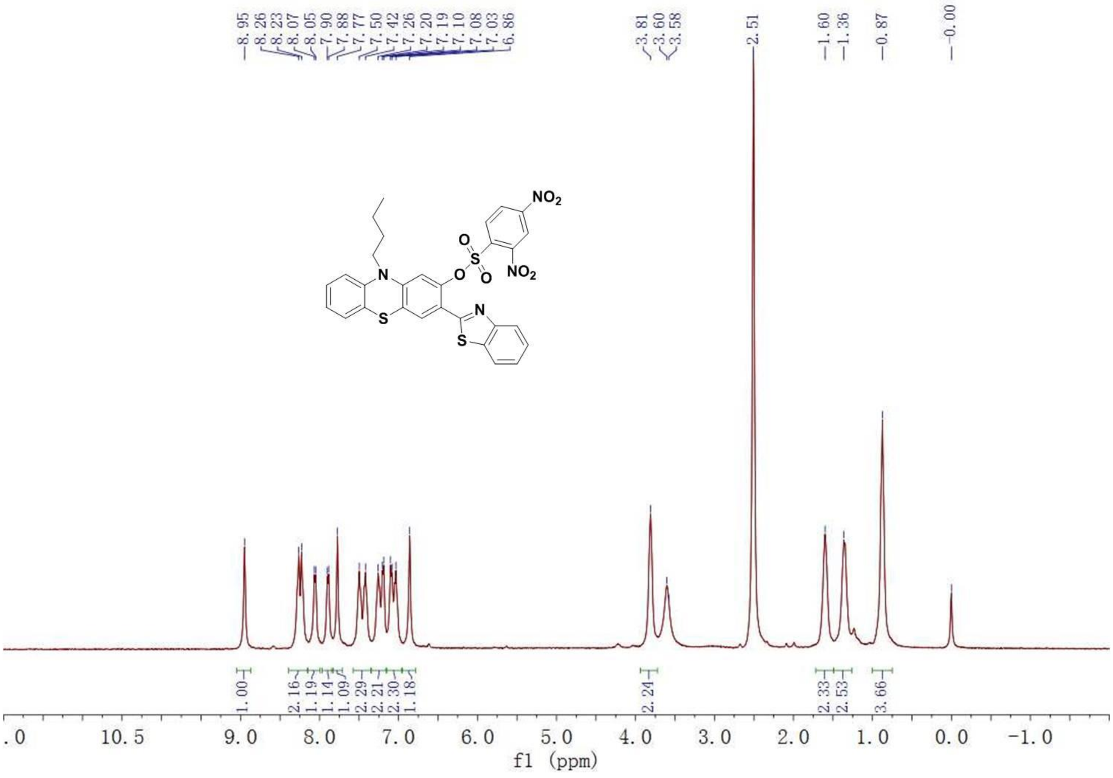

Figure S8 ¹H NMR spectrum of probe **1** in DMSO-d_6.

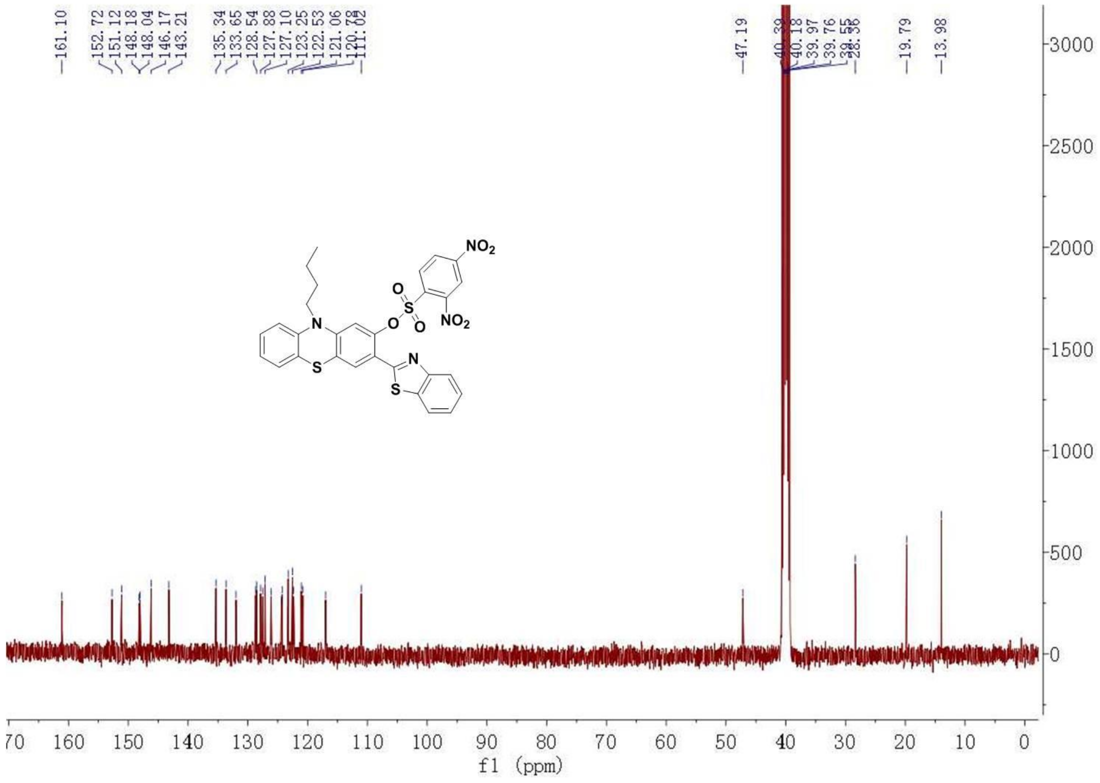

Figure S9 ^{13}C NMR spectrum of probe **1** in DMSO-*d*$_6$.

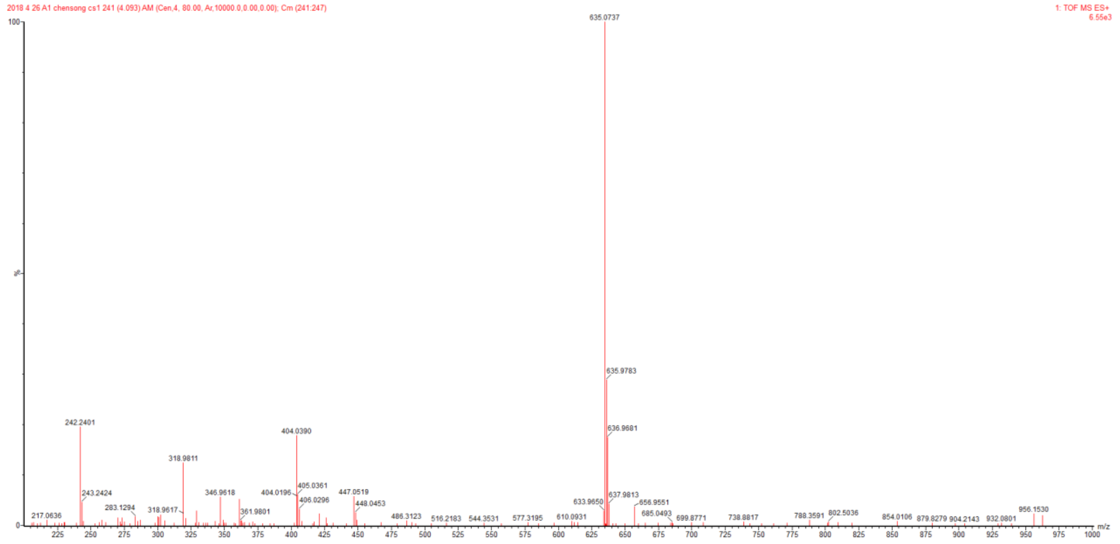

Figure S10 Mass spectrum of probe **1**.

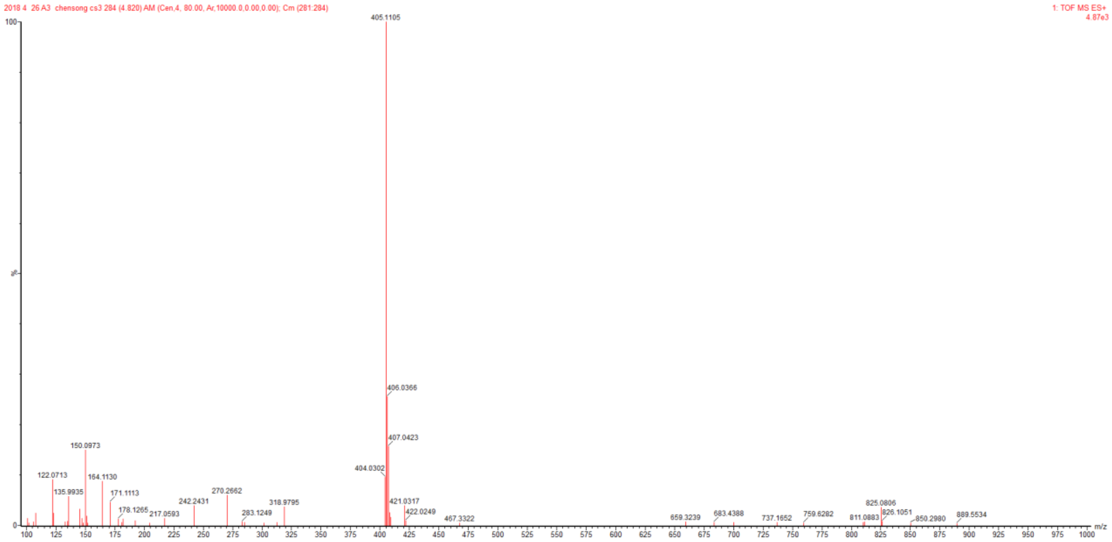

Figure S11 Mass spectrum of probe **1+Cys**.

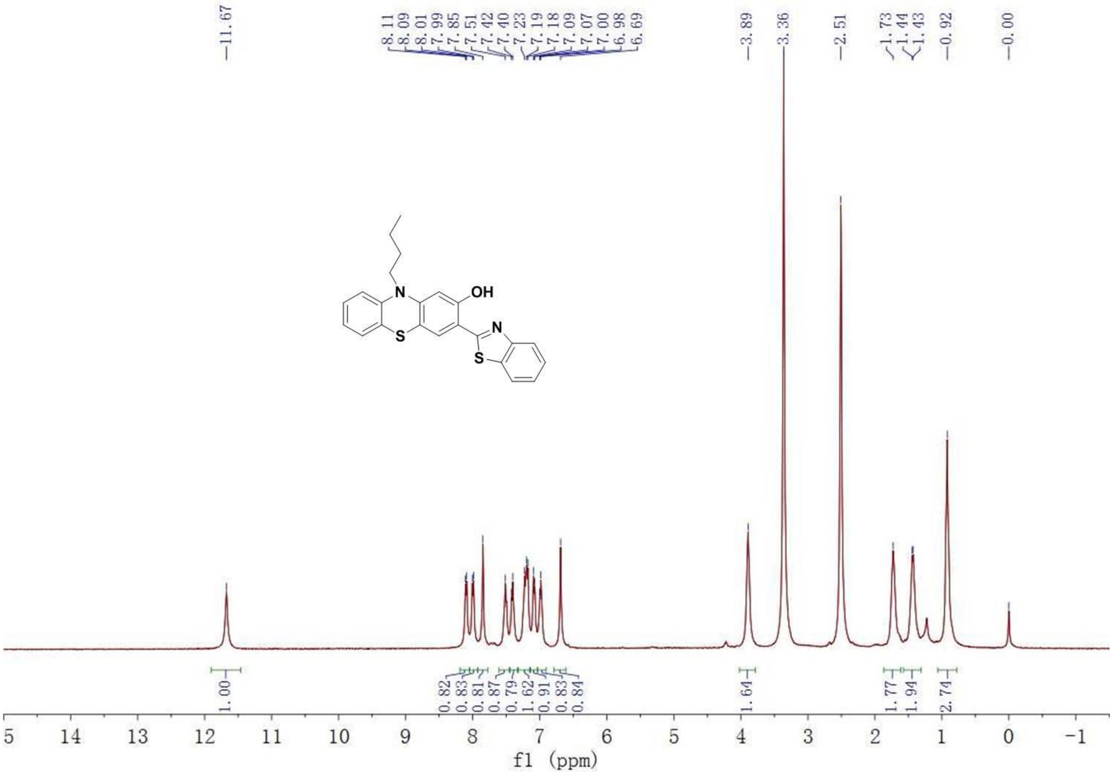

Figure S12 ^{1}H NMR spectrum of dye 2 in DMSO-d_6.

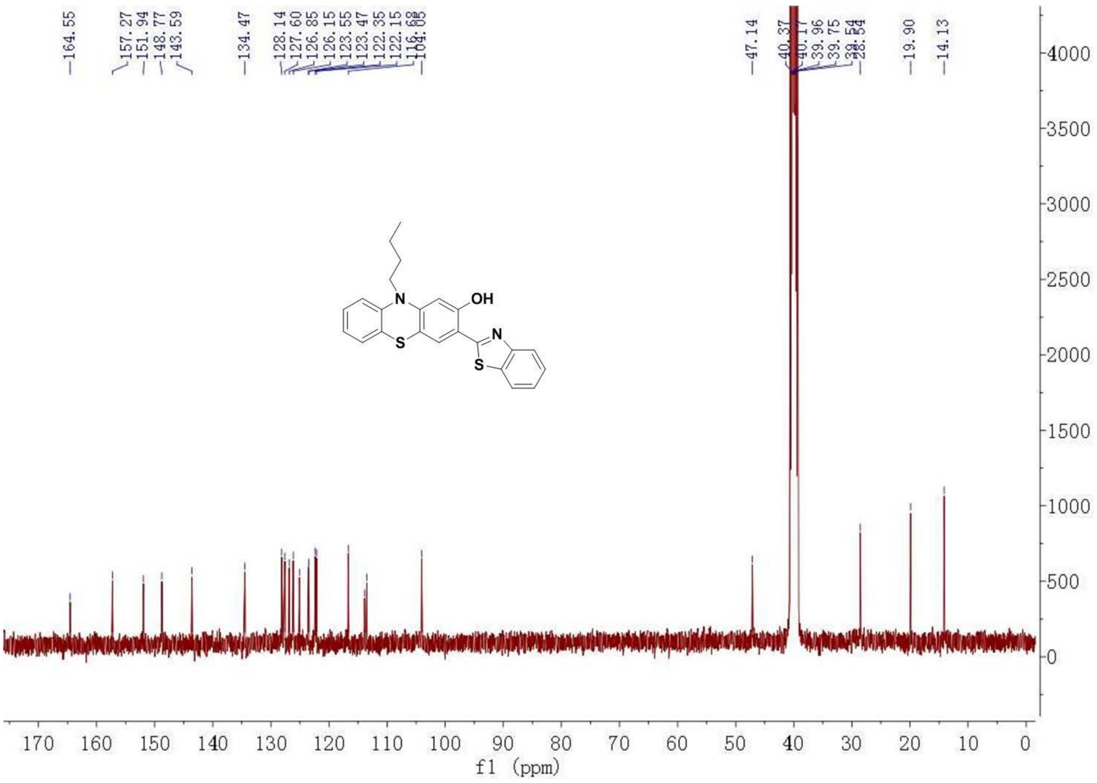

Figure S13 ^{13}C NMR spectrum of dye 2 in DMSO-d_6.

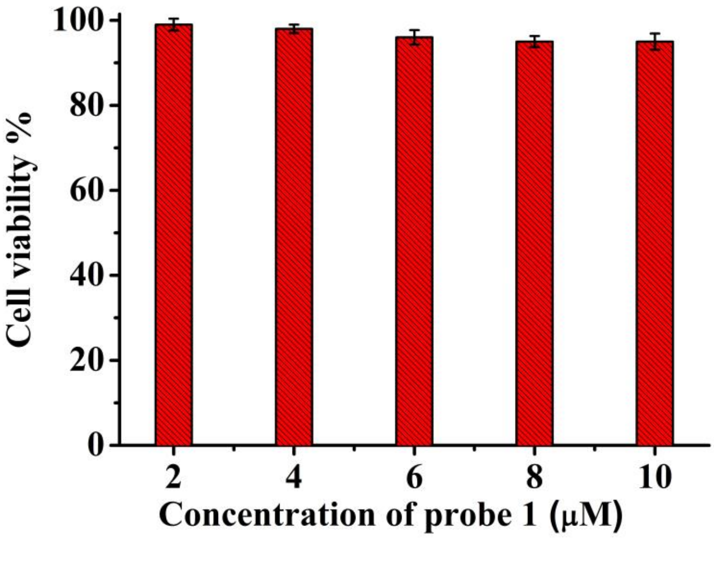

Figure S14 Percentage of viable A549 cells after treatment with different concentrations of probe 1 for 24 hours.

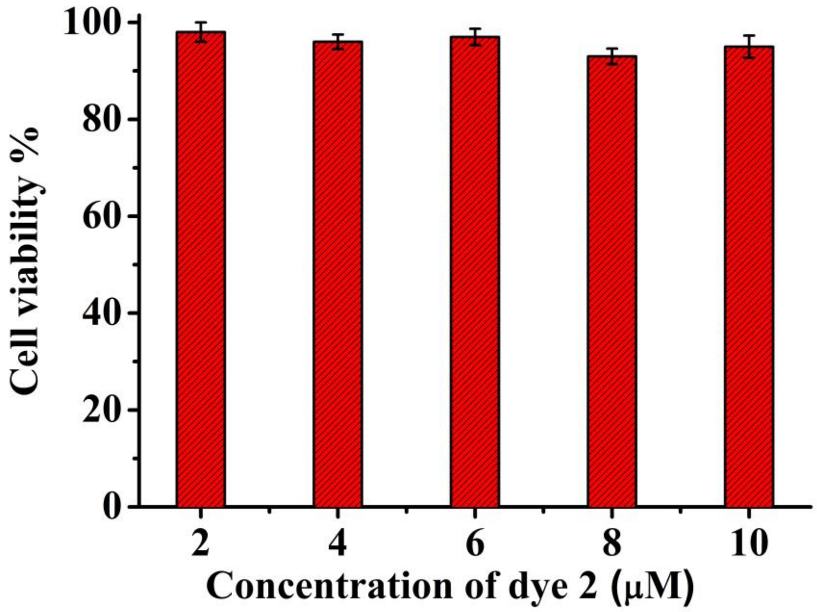

Figure S15 Percentage of viable A549 cells after treatment with different concentrations of dye 2 for 24 hours.

Table S1. Comparison of fluorescent probes for biothiols.

Probes	λex/λem (nm)	Stokes shift (nm)	Limit of detection	Response time	Reference
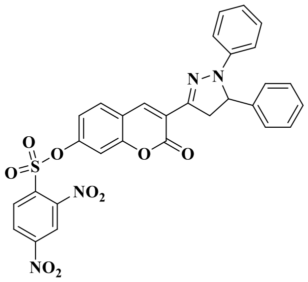	450/540	90	1.5× 10^{-8}M	10 min	Sensors actuat B-Chem, 2016, 223, 274-279.
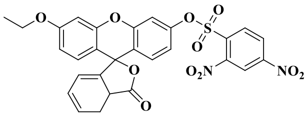	454/521	67	0.16 μM	10 min	Tetrahedron Letters, 2016, 57, 2478-2483
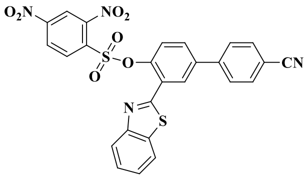	280/482	202	2.0× 10^{-8}M	20 min	Tetrahedron, 2017, 73, 589-593
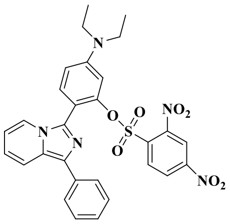	309/510	201	0.17 μM	10 min	Tetrahedron Letters, 2017, 58, 2654-2657.
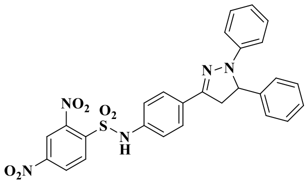	370/464	94	4.11× 10^{-7}M	12 h	Analyst, 2013, 138, 7169-7174.
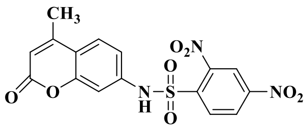	353/450	97	30 nM	2 h	Chem Commun, 2013, 49,4640-4642.
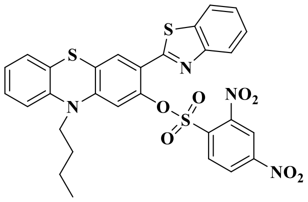	**413/530**	**117**	**0.12 μM**	**15 min**	**This work**
